# Rare earth and trace element signatures for assessing an impact of rock mining and processing on the environment: Wiśniówka case study, south-central Poland

**DOI:** 10.1007/s11356-016-7713-y

**Published:** 2016-09-25

**Authors:** Zdzisław M. Migaszewski, Agnieszka Gałuszka, Sabina Dołęgowska

**Affiliations:** Jan Kochanowski University, Świętokrzyska 15G, 25-406 Kielce, Poland

**Keywords:** REE enrichments, Metal(loid)s, Pyrite, Acid mine drainage, Rock mining impact

## Abstract

A detailed hydrogeochemical study was performed in the Wiśniówka mining area (south-central Poland). This covered three acid pit bodies, historic tailings acid ponds, acid pools, and additionally two neighboring rivers. All these acid mine drainage (AMD) waters are characterized by the pH in the range of 1.7 (pools) to 3.5 (tailings ponds). The most interesting is the Podwiśniówka acid pit lake that shows a very low pH (2.2–2.5) and very high concentrations of SO_4_^2−^ (2720–5460 mg/L), Fe (545–1140 mg/L), Al (86.2 mg/L), As (9603–24,883 μg/L), Co (1317–3458 μg/L), Cr (753–2047 μg/L), Cu (6307–18,879 μg/L), Ni (1168–3127 μg/L), and rare earth element (REE) (589–1341 μg/L). In addition, seeps that drain the Podwiśniówka mine tailings and partly aggregate piles form strong acid pools in the mining area. Along with these pools, in which As and REE contents reach 369,726 and 6288 μg/L, respectively, these waters are among the most distinctive As- and REE-rich AMD surface waters across the world. It is noteworthy that the Podwiśniówka acid pit lake and Wiśniówka Duża acid pit sump exhibit different element signatures and REE concentration patterns normalized to North American Composite Shale (NASC): the Podwiśniówka acid pit lake always shows a characteristic roof-shaped medium REE (MREE) profile with distinct enrichments in Gd, Eu, and Tb whereas the other one displays a step-shaped heavy REE (HREE) profile with positive Tb and Gd anomalies. The REE undergo fractionation during weathering and the subsequent leaching of dissolved and suspended fractions from rocks to acid water bodies where these and other elements are further fractionated by geochemical processes. This study shows that the individual REE have greater affinities for Mn, HREE for Fe and SO_4_^2−^, and only La and Ce for Al. This specific water geochemistry has enabled us to (i) pinpoint the location of AMD “hot spots” originated from quartzite mining and processing operations conducted by current and previous mining companies, (ii) predict the directions and effects of future strip mining for quartzites in the Wiśniówka Duża and Podwiśniówka open pits, and (iii) evaluate the potential impact of mining and processing effluents on the quality of rivers.

## Introduction

The origin, transport and fate of elements, including rare earth elements (REEs) and arsenic, in different acid mine drainage (AMD) sites have been studied for decades (e.g., Hudson-Edwards et al. 1999; Johannesson and Zhou 1999; Plumlee et al. 1999; Winterbourn et al. 2000; Worall and Pearson 2001; Druschel et al. 2004; Bednar et al. 2005; Gammons et al. 2005; Olías et al. 2006; Pérez-López et al. 2010; Nordstrom 2011; Grawunder et al. 2014; Migaszewski and Gałuszka 2015). These studies have also included geochemical processes that effect remobilization of trace and minor elements from sulfide ore and coal deposits, mineralized rock formations, and mine wastes to surface and underground waters (e.g., Reichenbach 1994; Monterroso and Macías 1998; Nordstrom 2011). This remobilization is initiated by oxidation of pyrite and iron-bearing sulfides in the presence of two natural oxidants, i.e., oxygen and subsequently at a lower pH by more effective ferric (Fe^3+^) iron (e.g., Garrels and Thompson 1960; Moses et al. 1987; Nordstrom and Alpers 1999a). This process is a potential source of acidification and dispersal of hazardous metal(loid)s into waters, sediments, soils, and biota (e.g., Aguilar et al. 2004; Younger et al. 2005; Cánovas et al. 2008; Martínez-Martínez et al. 2013). Moreover, this triggers a chain of reactions that lead to formation of secondary minerals (e.g., schwertmannite, ferrihydrite, goethite, lepidocrocite, hematite, hydroxy-green rusts, jarosite) that seem to play a decisive role in controlling the pH of pit pond or lake waters (e.g., Bingham et al.^1990^, ^1992^; Schwertmann et al. 1995; Acero et al. 2006). These complex processes induced primarily by anthropogenic activity are termed acid mine drainage. Because AMD waters jeopardize health of abiotic and biotic systems, the studies have also encompassed specific geochemical signatures to assess a metal(loid) pollution extent (Migaszewski et al. 2015).

The REEs have found an application in fingerprinting geologic and anthropogenic sources and in investigating geochemical processes that lead to fractionation of this element group in various environmental compartments, but particularly in surface and underground waters (e.g., Johannesson and Lyons 1995; Verplanck et al. 2004; Bozau et al. 2004; Gammons et al. 2005; Knappe et al. 2005; Leybourne and Johannesson 2008; Kulaksiz and Bau 2011; Migaszewski and Gałuszka 2015, 2016). In the surface water systems, the REE undergo fractionation between colloid/suspended matter and dissolved fractions (Ingri et al. 2000). The mobile REE fractions are very sensitive to small variations in the pH, redox potential, salinity, and concentrations of chelating agents, and participate in water-solid sorption-desorption, solution, and surface complexation involving inorganic and organic ligands (e.g., CO_3_^2−^, PO_4_^3−^, humic and fulvic acids), and coprecipitation with colloids and suspended matter. Of particular interest are acid AMD streams, ponds, and lakes that provide a direct insight into local and regional geologic sources highlighted by a number of specific REE profiles (e.g., Bozau et al. 2004; Verplanck et al. 2004; Grawunder et al. 2014; Migaszewski et al. 2015). The AMD waters commonly exhibit high concentrations of REE in the range of tens to hundreds micrograms per liter (Verplanck et al. 2001; Protano and Riccobono 2002; Gammons et al. 2005; Borrego et al. 2012), and different metal(loid)s, especially Fe and As. The mentioned last reflects the fate of other trace elements in AMD aqueous environments. Arsenic undergoes geochemical interactions within the water-colloid-ochreous precipitate system. Weathering of pyrite releases As which is trapped by unstable schwertmannite. The transformation of this mineral into ferrihydrite at the pH of 2.5–4.5 and subsequently into goethite at the pH of 3–7 leads to desorption of As. The decrease in the pH brings about reductive dissolution of iron oxyhydroxides triggering also the release of As (e.g., Bingham et al. 1990, 1992; Smedley and Kinniburgh 2002; Bednar et al. 2005; Courtin-Nomade et al. 2005).

**Fig. 1:**
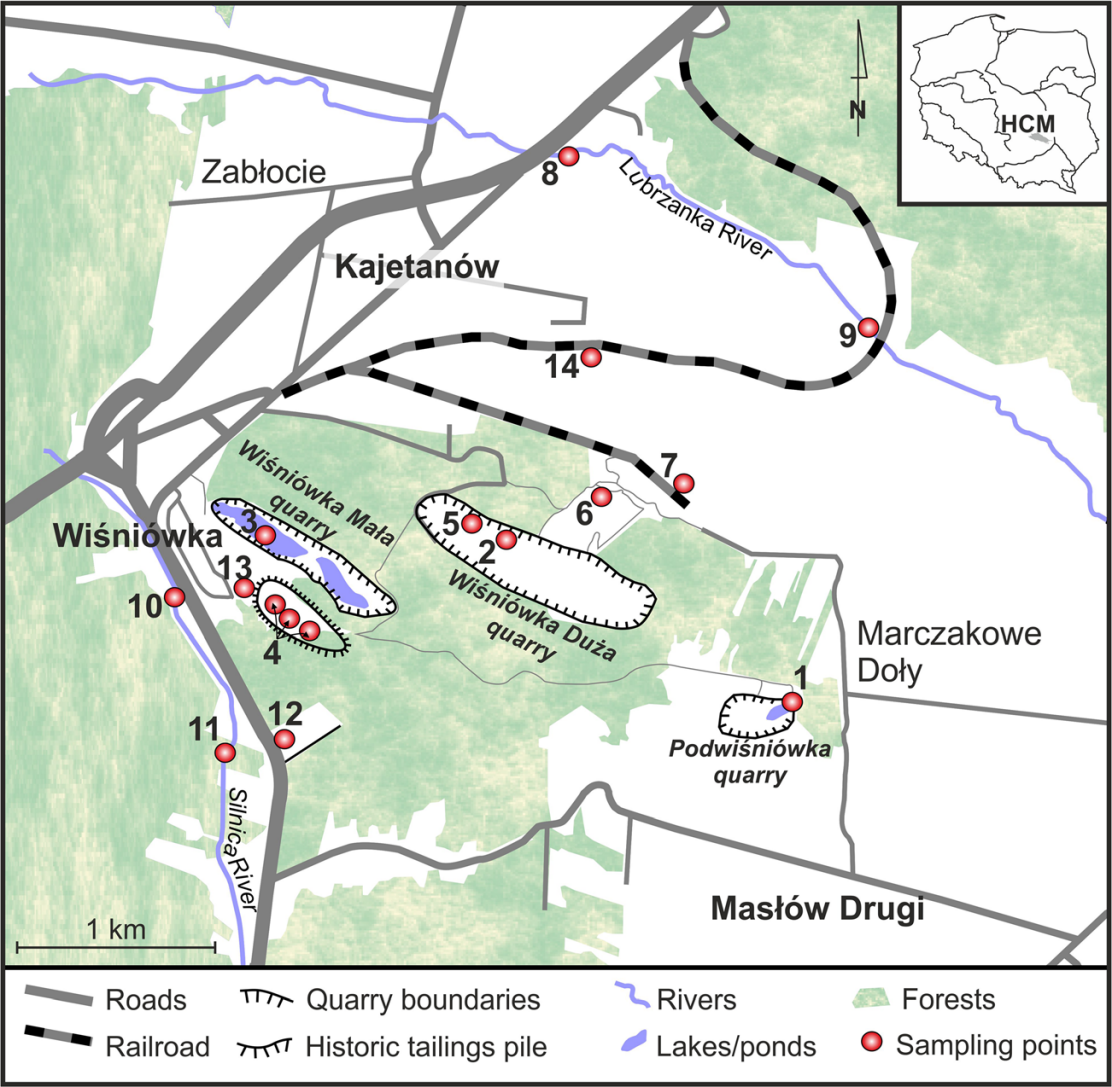
Location of study area with sampling sites. Explanations: *1*, Podwiśniówka lake; *2*, Wiśniówka Duża pit sump pond; *3*, Wiśniówka Mała lake; *4*, historic tailings pile ponds; *5*, Wiśniówka Duża pool; *6*, Wiśniówka aggregate processing plant pool; *7*, Wiśniówka aggregate processing plant pool; *8*, Lubrzanka River upstream; *9*, Lubrzanka River downstream; *10*, Silnica River upstream; *11*, Silnica River downstream; *12*, culvert under highway E-73; *13*, culvert under housing development; *14*, culvert under railroad track in Kajetanów

**Fig. 2:**
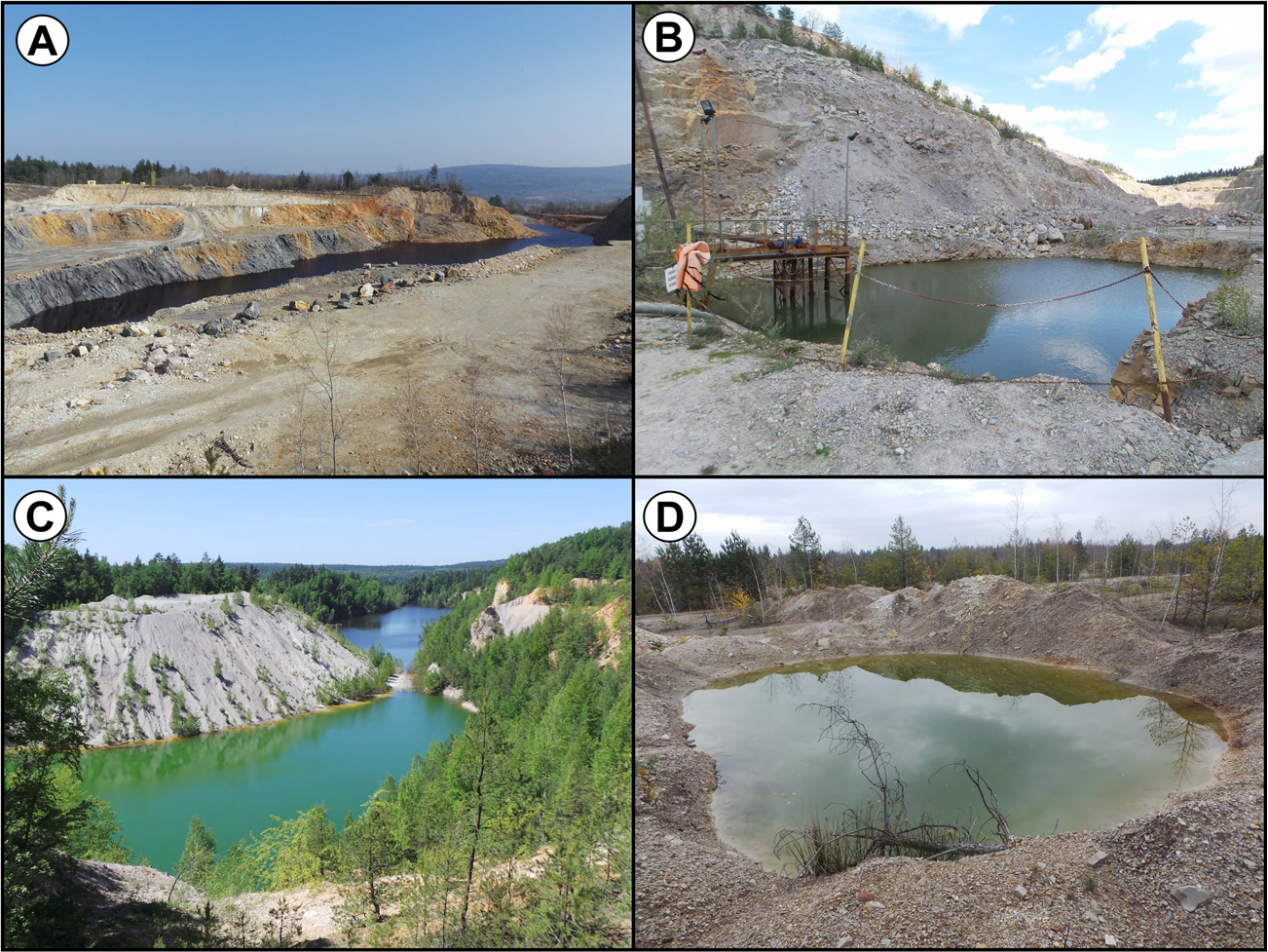
A general view of **a** Podwiśniówka acid pit lake, **b** Wiśniówka Duża pit sump, **c** Wiśniówka Mała acid pit lake, **d** historic tailings acid pond

The previous studies conducted during 2006–2012 encompassed only two water bodies, i.e., the existing Wiśniówka Mała acid pit lake (Fig. 1) and the former Podwiśniówka acid pit pond. Their purpose was to identify geochemical processes of AMD water generation based on determinations of δ^34^S-FeS_2_, δ^34^S-SO_4_^2−^, δ^18^O-SO_4_^2−^, δ^18^O-H_2_O, and δD-H_2_O and selected trace metal(loid)s (Migaszewski et al. 2007a, 2008, 2009, 2013, 2014). In addition, the scope of investigation also covered mineralogical and petrological examinations of pyrite mineralization zone exposed on the western wall of the “Podwiśniówka” quarry (Migaszewski et al. 2007b; Migaszewski and Gałuszka 2010). The situation changed when the new mining company started intensive quartzite extraction in the Podwiśniówka quarry. This operation lasted from the spring to the fall of 2014. Deep quarrying for quartzites along the pyrite-bearing zone has induced generation of AMD waters, leaving an acid pit lake on the lowest mining bench (Fig. 2a). This water shows totally different chemistry highlighted by much higher levels of sulfates, arsenic, and trace metals compared to those recorded in the former shallow acid pit pond. To make this picture worse, formation of strongly As- and metal-concentrated acid pools near tailings piles and the use of water from the abandoned Wiśniówka Mała pit lake for washing of crushed quartzite aggregate from the Podwiśniówka quarry have posed an additional potential threat to the environment.

The principal objectives of the study presented here are to (i) determine the current hydrogeochemistry of AMD water bodies, (ii) apply characteristic element profiles to pinpoint rock mining and processing operations, and (iii) evaluate AMD water influence on the neighboring surface water systems.

## Materials and methods

### Study area location and geologic framework

The present study covered a large Wiśniówka mining area located about 5 km north of Kielce, the capital of the Holy Cross Mountains region (south-central Poland). The study area lies within a dismembered massif (elevation of 455.7 m above sea level (a.s.l.)) assigned to the western part of the Main Range of the Holy Cross Mountains. The Wiśniówka Massif is characterized by highly complicated tectonics with intense folding and faulting and very complex stratigraphy, being the subject of controversy and dispute among geologists for decades (Żylińska et al. 2006; Konon 2008, and references therein). There are three quarries occurring in the mining area (from the west to the east): Wiśniówka Mała, Wiśniówka Duża, and Podwiśniówka (Fig. 1). The basic lithostratigraphic unit of the Wiśniówka Massif is the Wiśniówka Sandstone Formation (Orłowski 1975) that consists of thick-bedded Upper Cambrian (Furongian) quartzites and quartzitic sandstones (fine-grained quartz arenites) interbedded with quartzitic siltstones, clayey shales and subordinate bentonites and tuffites, in places with pyrite mineralization zones (Jaworowski and Sikorska 2006).

These pyritiferous zones, which are fully exposed only in the Podwiśniówka and partly in the Wiśniówka Duża quarries, are a source of AMD water generation. The Podwiśniówka quarry is distinguished by specific mineralogical features of predominant crypto- to micro-grained As-rich pyrite (gel-pyrite) that favor its rapid oxidation as evidenced by the previous studies (Migaszewski et al. 2007a, 2008). Moreover, in the northern part of this quarry, hematite and goethite mineralization zone crops out at the surface. There are xenoliths of pyritiferous quartzites within this iron(III) oxide and oxyhydroxide zone. It is noteworthy that hydrothermal REE-bearing minerals (gorceixite, florencite, xenotime) occur primarily within clayey shale and subordinate tuffite interbeds of the Podwiśniówka quarry as evidenced by the previous studies (Migaszewski et al. 2007b; Migaszewski and Gałuszka 2010).

Consequently, there are numerous acid water bodies in the mining area showing different chemistry. These include Podwiśniówka acid pit lake, Wiśniówka Mała acid pit lake, Wiśniówka Duża pit sump, small ponds on the surface of historical tailings piles, and periodic highly acidic and metal(loid) concentrated pools that drain mine and processing tailings piles or exposed pyrite-rich zones where the landscape was stripped for mining (Fig. 2a–d). Of all the examined acid water bodies, the most interesting is the Podwiśniówka acid pit lake that takes up an area of over 1 ha with an average depth of about 2.3 m (Figs. 1 and 2a). Its water level coordinate is approx. 399.0 m a.s.l., and its cubature is about 23,600 m^3^. The Wiśniówka Duża pit sump has in turn an area of about 260 m^2^ and a depth of about 5.4 m (Figs. 1 and 2b) whereas Wiśniówka Mała pit lake covers an area of about 5.2 ha with an average depth of about 11 m (Figs. 1 and 2c). The coordinates of their water levels are 367.7 and 355.7 m a.s.l., respectively. During over 100 years of intermittent quarrying, pyrite-bearing waste material was stockpiled on a few hectares of land close to the southwestern boundary of Wiśniówka mining area (no. 4 in Fig. 1). The mining area borders on two rivers: Lubrzanka to the north and Silnica to the west (Fig. 1).

### Field geochemical measurements and sampling

Fieldwork was conducted from July 14 of 2014 through March 30 of 2016. This included sampling of AMD waters (35 samples) and additionally river waters (15 samples) for chemical analysis. Sampling, which is a crucial stage of each environmental study, was carried out according to US EPA recommendations (ISO/IEC:17025: 2005; NORDIC INNOVATION CENTRE 2007). All the water samples for trace element measurements were filtered through 0.45-μm pore-sized polytetrafluoroethylene (PTFE) syringe filters and placed in 50-mL polypropylene vials. The filtered and untreated water samples were transported to the Geochemical Laboratory of the Institute of Chemistry, Jan Kochanowski University, in Kielce on the same day and stored in a refrigerator at a temperature of about 4–6 °C.

Fieldwork also included direct measurements of pH, electric conductivity (EC), total dissolved solids (TDS), and temperature (T) of water, using a manual microprocessor pH/Eh-meter SP300 with a combination pH-electrode probe Winlab and a manual microprocessor EC-meter SC300 with a four-electrode combined conductivity probe WinLab (both Slandi, Poland) equipped with temperature sensors. In addition, alkalinity (CaCO_3_ mg/L), and concentrations of SO_4_^2−^, PO_4_^3−^, and total Fe were determined on-site using a field spectrophotometers LF-205 and LF-300 Slandi, Poland.

During sample collection, transport, storage, and preparation, procedures were followed to minimize the possibility of contamination. A set of water samples included one blank (deionized water from the laboratory that was processed in the field along with the environmental samples) and one replicate sample for each sampling series.

### Sample preparation and chemical analysis

All the samples were analyzed for Al, As, Cd, Co, Cr, Cu, Mn, Ni, Pb, Tl, and Zn, using an Inductively Coupled Plasma-Quadropole Mass Spectrometer (ICP-QMS; model ELAN DRC II, Perkin Elmer). Operating conditions were as follows: sweeps/reading, 20; readings/replicate, 3; replicates, 4; nebulizer gas flow, 0.99 L/min; plasma gas flow, 1.5 L/min; lens voltage, 6.50–6.75 V; plasma power, 1275 W. The ICP-QMS instrument was optimized with a standard daily procedure. For trace element determinations, a set of Multielement Calibration Standards Perkin Elmer solutions were applied. Quantification of REE was done with an external standard method. Working solutions with REE concentrations in the range of 0.1–10 μg/L were used to construct the four-point calibration curve (*r* ^2^ > 0.999). These solutions were prepared using the multielement standard solution from PerkinElmer (10 mg/L REE in 2 % nitric acid). Dilution of standards and samples was done with 2 % nitric acid prepared from ultrapure HNO_3_ (65 %, Merck) and distilled water. Iridium at a concentration of 100 μg/L (prepared from Perkin-Elmer single-element standard solution containing 1000 mg/L Ir) was applied as an internal standard. Physical interferences were eliminated by the use of internal standard and sample dilution (10–100 times, depending on concentrations of total dissolved solids). Spectral interferences were eliminated by application of the following correction equations:

$$ \begin{array}{c}\hfill {}^{142}Nd{:}^{142}Nd=-0.125653\ {\times}^{140}Ce\hfill \\ {}\hfill {}^{149}Sm{:}^{149}Sm=-0.012780\ {\times}^{157}Gd\hfill \\ {}\hfill {}^{160}Gd{:}^{160}Gd=-0.004016\ {\times}^{163}\mathrm{Dy}\hfill \\ {}\hfill {}^{164}\mathrm{Dy}{:}^{164}\mathrm{Dy}=-0.047902\ {\times}^{166}Er\hfill \\ {}\hfill {}^{172}Yb{:}^{172}Yb=-0.005865\ {\times}^{178}Hf\hfill \end{array} $$

The standard reference materials (SRMs) used for measuring element concentrations in the samples were NIST 1643e (trace elements in water) and the geologic multielement reference material (GM-ERM) PPREE1 (Table 2 in Verplanck et al. (2001)) for waters. Quality control included both accuracy (SRM) and precision (triplicates). The average recovery of elements from the SRM was 80–110 %, and the uncertainty of the method was below 10 %. The relative standard deviation (RSD) values were <4 % for most of the analyzed samples.

### REE ratios and enrichments

For the purpose of this study, the REEs (= lanthanides) were divided into two conventionally termed subgroups: (i) light REE (LREE) including La through Eu and (ii) heavy REE (HREE) from Gd through Lu, respectively. The third discriminated subgroup, medium REE (MREE; Sm through Ho) partly overlaps LREE and HREE. In order to compare REE concentrations in different water bodies of the Wiśniówka mining area, the results derived from these element measurements were normalized to North American Shale Composite (NASC) (Haskin et al. 1968; Gromet et al. 1984). This eliminates the characteristic “zigzag” REE distribution patterns and enables us to identify any individual REE anomalies in various geologic and environmental materials (Migaszewski and Gałuszka 2015).

The Ce/Ce_NASC_^*^ ratio in the Wiśniówka AMD waters was calculated from the Eq. (1) (Bau and Dulski 1996) and reported in Table 4:

1 $$ Ce/{Ce_{\mathrm{NASC}}}^{*} = {Ce}_{\mathrm{NASC}}/\left(0.5{La}_{\mathrm{NASC}}+0.5{ \Pr}_{\mathrm{NASC}}\right) $$

where Ce_NASC_^*^ is a background concentration whereas La_NASC_ and Pr_NASC_ are the NASC-normalized La and Pr concentrations, respectively.

The same equation structure was consistently employed for Eu/Eu_NASC_^*^, Gd/Gd_NASC_^*^, and Tb/Tb_NASC_^*^ ratios. However, due to distinct enrichment of Gd, it was inadmissible to use this element in calculation of Eu anomaly. This is the reason why modification of equation had to be made (Gd was substituted for Dy). The Eu/Eu_NASC_^*^ ratio in all the examined acid waters was calculated from the Eq. (2) (German et al. 1991, modified by the present authors) and given in Table 4:

2 $$ Eu/{Eu_{\mathrm{NASC}}}^{*} = {Eu}_{\mathrm{NASC}}\Big)/\left(\left({Sm}_{\mathrm{NASC}} + {\mathrm{Dy}}_{\mathrm{NASC}}\right)/2\right) $$

Similarly, because of distinct enrichment of Tb, this element was not used in calculation of Gd anomaly. The Gd/Gd_NASC_^*^ ratio was computed from the Eq. (3) and presented in Table 4:

3 $$ Gd/{Gd_{\mathrm{NASC}}}^{*} = {Gd}_{\mathrm{NASC}}/\left(\left({Sm}_{\mathrm{NASC}} + {\mathrm{Dy}}_{\mathrm{NASC}}\right)/2\right) $$

The Tb/Tb_NASC_^*^ ratio was in turn computed from Eq. (4) and presented in Table 4:

4 $$ \mathrm{Tb}/{{\mathrm{Tb}}_{\mathrm{NASC}}}^{*} = {\mathrm{Tb}}_{\mathrm{NASC}}\Big)/\left(\left({Sm}_{\mathrm{NASC}} + {\mathrm{Dy}}_{\mathrm{NASC}}\right)/2\right) $$

where Eu/Gd/Tb_NASC_^*^ are background concentrations whereas Sm_NASC_ and Dy_NASC_ are the NASC-normalized Sm and Dy concentrations, respectively.

Values below 0.8 are indicative of negative anomalies whereas those above 1.2 point to positive anomalies (Grawunder et al. 2014). In addition, the La_NASC_/Yb_NASC_ and Sm_NASC_/Yb_NASC_ ratios were computed to assess depletion (<0.8) or enrichment (>1.2) of distinguished REE subgroups: LREE (La), MREE (Sm), and HREE (Yb).

### Statistical analysis

Data processing included the Microsoft Excel application for summary statistics and graphical presentation of REE patterns, and cluster and factor analysis. In the statistics calculations, the censored (below lower limit of determination) values were replaced with arbitrary values equal to 50 % of their determination limit. Both cluster and factor analysis is a multivariate analysis. The former enables us to form groups of related variables. Based on similarities and dissimilarities within and between classes, the objects are divided into “clusters.” The data obtained were normalized and standardized using STATISTICA Base software (StatSoft Inc.), with the linkage distances for a particular case divided by the maximal linkage distance (D_link_/D_max_). The data derived from this study were originally centered by Box-Cox function with all variables standardized later. The cluster analysis was done using the Ward’s method with a square Euclidean distance as a measure of similarity. The Ward’s method enables an analysis of variance approach to evaluate the distances between the clusters in order to minimize the sum of squares of any two clusters (that can be formed at each step).

The factor analysis in turn allows us to describe variability among the dataset. This groups similar variables into specific structures without distinguishing between dependent and independent variables. In the present study, two main factors were retained (explained variance: F1 14.01; F2 12.94). The first factor is loaded by pH, EC, As, Co, Cu, Fe, Ni, Sc, and SO_4_^2−^ and the other by Al, Ce, Cd, La, Nd, Pr, and Zn. With regard to other variables, cross-loading is observed. The scatterplot (not included for the sake of brevity) obtained does not depict the independent factors.

### Petrographic study

The principal objective of the preliminary petrographic study was to find “signatures” in rock-water interactions. The ore and gangue minerals and rocks of the Wiśniówka Duża and additionally Podwiśniówka quarries were examined using stereoscopic and petrographic microscopes (Leica M205 A, Nikon LV 100 Pol with CITL Cathodoluminescence module MK5-2) and a scanning electron microscope (SEM) LEO 1430 (signal A = SEI, and BEI, magnification = ×1310–1850, EHT = 15.00 kV, WD = 16–19 mm). Semi-quantitative chemical analysis of pyrite was done with an EDS ISIS Detector (Oxford Instruments Ltd.). For this purpose, two standards, i.e., 30. Pyrite and 19. Skutterudite (SPI Standards 53 Minerals 02753-AB, West Chester, PA 19381-0656, USA), were used. This petrographic study was performed on raw and polished rock pieces (stereoscopic microscope), polished thin sections (polarizing microscope), and carbon-coated polished thin sections (SEM).

In addition, a few samples of pyrite were analyzed for trace metal(loid)s using the CETAC LSX 500+ laser ablation module coupled with the Perkin Elmer ICP-QMS ELAN DRC II instrument. The CETAC laser ablation module was equipped with the Q-switched Nd:YAG laser, frequency quadrupoled to 266 nm. The single-line raster LA method was used. Sample surfaces were ablated with 250 shots at pulse energy of >9 mJ. The other optimized laser parameters included a pulse repetition rate of 10 Hz, a delay between spots 2 min, and a spot diameter of 25 μm. A time-resolved plot of signal for each element was integrated, and concentrations were computed using DigiLaz™-II operating software by CETAC. Operating conditions for the Perkin Elmer ICP-MS/MS ELAN DRC II instrument were as follows: low resolution, power (∼1600 W), and sample gas flow rate (415 ± 7 kPa at 20 l/min Ar) adjusted to maximize atomic ion signal. The matrix-matched USGS microanalytical reference material, i.e., polymetal sulfide MASS-1, was used for quantitative analysis. The samples were analyzed for seven elements: As, Cd, Co, Cu, Ni, Pb, and Zn.

## Results and discussion

### Geochemistry of waters

The mean values of selected physicochemical parameters and the mean concentrations of sulfates, phosphates, REEs, and trace elements in the AMD and river waters from the Wiśniówka area are summarized in Tables 1, 2 and 3. It should be underscored that due to the use of 0.45-μm pore-sized filters, these contents reflect both true dissolved and partly colloidal element species.

**Table 1:** The mean values of measured physicochemical and chemical parameters in the Wiśniówka AMD water bodies

Parameter	Podwiśniówka pit lake (*N* = 6)	Wiśniówka Duża pit sump (*N* = 5)	Wiśniówka Mała pit lake (*N* = 5)	Historic tailings pile ponds (*N* = 6)	Maximum allowable limits MEP (2014)
pH	*2.2–2.5*	*3.0–3.4*	*3.0–3.1*	*3.1–3.5*	*6.5–9.0*
Eh (mV)	266 ± 6	222 ± 11	232 ± 4	211 ± 12	n.d.
EC (mS/cm)	5.57 ± 0.57	1.15 ± 0.12	1.10 ± 0.14	1.08 ± 0.45	n.d.
TDS (mg/L)	2790 ± 288	574 ± 63	528 ± 60	542 ± 221	n.d.
SO_4_^2−^ (mg/L)	*3978 ± 885*	*575 ± 184*	*534 ± 64*	*608 ± 360*	*500*
PO_4_^3−^ (mg/L)	7.92 ± 0.63	0.04	0.17	0.01 ± 0.008	n/a
Alkal. (mg/L)	75 ± 23	119 ± 70	65 ± 40	88 ± 68	n/a
Fe_total_ (mg/L)	860 ± 260	9.1 ± 4.4	23.3 ± 10.0	5.2 ± 4.4	*10*
Al (mg/L)	*86.02*	*18.9*	*25.5*	*10.1 ± 10.0*	*3*
As (μg/L)	*15,416 ± 5197*	15.1 ± 11.4	82.4 ± 52.7	47.5 ± 38.7	*100*
Cd (μg/L)	3.80 ± 0.94	0.52 ± 0.13	0.68 ± 0.10	0.77 ± 0.32	400 (200)^a^
Co (μg/L)	*2413 ± 776*	200 ± 66	135 ± 24	135 ± 64	*1000*
Cr (μg/L)	*1377 ± 413*	41 ± 20	76 ± 14	61.8 ± 60.6	*500*
Cu (μg/L)	*14,314 ± 5278*	*1923 ± 619*	451 ± 111	446 ± 348	*500*
Mn (μg/L)	1922 ± 1349	1064 ± 536	6317 ± 1606	1871 ± 343	n.d.
Ni (μg/L)	*2303 ± 783*	207 ± 75	188 ± 5	243 ± 123	*500*
Pb (μg/L)	4.86 ± 3.91	0.68 ± 1.12	1.01 ± 1.32	0.91 ± 0.96	500
Tl (μg/L)	7.63 ± 2.10	0.72 ± 0.40	0.38 ± 0.10	0.08 ± 0.02	1000
Zn (μg/L)	419 ± 293	213 ± 163	142 ± 30	215 ± 33	2000
Sc (μg/L)	310 ± 94	44.6 ± 22.5	14.2 ± 5.3	19.0 ± 25.3	n/a
Y (μg/L)	266 ± 74	109.2 ± 13.3	40.1 ± 18.1	111.3 ± 78.7	n/a
La (μg/L)	82 ± 23	11.40 ± 2.96	9.43 ± 2.74	13.6 ± 7.5	n/a
Ce (μg/L)	260 ± 80	35.9 ± 9.9	25.2 ± 8.1	49.5 ± 30.8	n/a
Pr (μg/L)	41 ± 12	6.18 ± 1.90	3.69 ± 1.29	8.7 ± 6.1	n/a
Nd (μg/L)	216 ± 62	34.6 ± 9.8	17.9 ± 6.6	48.1 ± 34.9	n/a
Sm (μg/L)	92 ± 29	12.76 ± 3.90	7.19 ± 2.64	16.03 ± 11.93	n/a
Eu (μg/L)	24.5 ± 6.7	3.19 ± 1.13	1.94 ± 0.86	4.26 ± 3.13	n/a
Gd (μg/L)	113.3 ± 29.2	20.3 ± 6.4	10.5 ± 4.0	23.8 ± 17.6	n/a
Tb (μg/L)	15.6 ± 4.1	3.28 ± 1.03	1.57 ± 0.69	3.80 ± 2.71	n/a
Dy (μg/L)	74.0 ± 19.2	19.4 ± 6.3	8.25 ± 3.82	20.9 ± 15.3	n/a
Ho (μg/L)	12.5 ± 3.2	3.85 ± 1.24	1.49 ± 0.71	3.96 ± 2.87	n/a
Er (μg/L)	30.6 ± 7.9	10.68 ± 2.91	3.80 ± 1.78	10.4 ± 7.6	n/a
Tm (μg/L)	3.74 ± 1.10	1.50 ± 0.38	0.49 ± 0.25	1.29 ± 0.94	n/a
Yb (μg/L)	24.40 ± 6.14	9.31 ± 2.39	2.98 ± 1.62	8.06 ± 6.08	n/a
Lu (μg/L)	3.54 ± 1.01	1.37 ± 0.40	0.44 ± 0.25	1.16 ± 0.89	n/a
∑REE (μg/L)	992.88 ± 281.99	173.67 ± 50.07	94.85 ± 35.19	213.57 ± 147.41	
LREE (μg/L)	715.26 ± 211.18	103.95 ± 29.36	63.31 ± 22.18	140.21 ± 94.18	
HREE (μg/L)	277.62 ± 71.64	69.72 ± 20.91	29.54 ± 13.12	73.37 ± 53.79	
MREE (μg/L)	331.95 ± 91.03	55.55 ± 31.30	22.72 ± 7.02	72.74 ± 53.26	

Element and sulfate concentrations, and pH values exceeding maximum allowable limits (acc, to the Regulation of the Ministry of Environmental Protection, Poland, 2014) are italicized

*Alkal.* alkalinity (CaCO_3_ mg/L), *n.d.* not determined, *n/a* not applicable

^a^Daily (monthly) discharge

**Table 2:** The pH values and concentrations of REE, sulfates, and selected trace elements in acid water bodies of the Wiśniówka mining area vs. other AMD sites showing high As and REE concentrations

Localization	pH	SO_4_^2−^	Fe_total_	∑REE	As	Co	Cr	Cu	Ni
		mg/L	μg/L						
Podwiśniówka acid pit lake^a^	2.4	3978	860	993	15,416	2413	1377	14,314	2303
Wiśniówka Duża quarry (acid pool no. 5)	1.8	36,250	5800	2433	369,726	1814	2321	8742	4768
Wiśniówka processing plant (acid pool no. 6)	2.8	91,800	950	6288	16,721	2776	1349	6090	2936
Wiśniówka processing plant (acid pool no. 7)	1.7	26,950	6572	4306	288,850	5100	15,850	19,000	9475
Serwis acid pool P1/1, Poland^b^	2.6	n.d.	4.6	1066	<0.01	700	10	0.2	200
Richmond Mine, Iron Mountain, USA^c^	n.d.	n.d.	68,100	n.d.	850,000	3.6	2.6	9800	6.3
Tailings Stock, Carnoulés, France^d^	∼1.2	17,831	4355	n.d.	638,000	n.d.	n.d.	n.d.	n.d.
Reigous Creek, Carnoulés, France^d^	4.3	4336	1424	n.d.	264,000	n.d.	n.d.	n.d.	n.d.
Corta Atalaya acid pit lake, Spain^e^	1.2	41,900	36,675	n.d.	158,730	18,689	1295	1,350,000	5214
Rio Tinto River, Spain^f^	1.4	15,000	4300	n.d.	22,000	n.d.	n.d.	210,000	n.d.
Koehler Breakdown, USA^g^	2.8	2230	493	n.d.	8039	n.d.	n.d.	n.d.	n.d.
Virginia Canyon Mine, USA^g^	2.8	2940	381	n.d.	3687	n.d.	n.d.	n.d.	n.d.
Upper Rio Agrio, Patagonia, Argentina^h^	1.6	14,700	2650	1637	4780	n.d.	n.d.	n.d.	n.d.
Santa Lucia (mine effuents), sample L2^i^	2.7	6399	4330	370	191	n.d.	n.d.	95	n.d.
Santa Lucia (mine effuents), sample L3^i^	2.5	2149	264	860	5	n.d.	n.d.	1790	n.d.
Paradise Portal (PPREE1), CO, USA^j^	5.3	1250	627	458	5	100	<45	5	19

*n.d.* not determined

^a^Average of six measurements

^b^Migaszewski et al. (2015)

^c^Nordstrom. Alpers (1999b)

^d^Giloteaux et al. (2012)—Tailings Stock 108,000–638,000 μg/L; Reigous Creek 179,000–264,000 μg/L

^e^España et al. (2008)

^f^Hudson-Edwards et al. (1999)

^g^Bednar et al. (2005)

^h^Gammons et al. (2005)

^i^Romero et al. (2010)

^j^Verplanck et al. (2001)

**Table 3:** Average measured concentrations of REE, trace elements and sulfates vs. pH and alkalinity in the Silnica and Lubrzanka rivers adjacent to the Wiśniówka mining area

Localization	pH	∑REE	SO_4_^2−^	Alkal.	Fe_total_	As	Co	Cr	Cu	Ni
		μg/L	mg/L	mg/L	mg/L	µg/L	µg/L	µg/L	µg/L	µg/L
Silnica R.^a^ (no. 10) (*N* = 3)	7.4	0.883	54	135	0.7	3.02	0.60	3.21	6.1	5.2
Silnica R.^b^ (no. 11) (*N* = 4)	7.2	1.160	61	167	2.8	4.86	12.48	3.06	13.18	20.0
Silnica R.^a^ (no. 10) (March 14, 2016)	7.3	0.695	43	49	0.2	1.00	1.66	2.61	7.0	3.0
Silnica R.^b^ (no. 11) (March 14, 2016)^c^	6.9	5.625	134	13.9	0.4	2.25	30.9	1.79	86	31.8
Lubrzanka R.^a^ (no. 8) (*N* = 2)	7.8	0.618	38	278	0.59	1.09	0.18	4.61	6.10	3.91
Lubrzanka R.^b^ (no. 9) (*N* = 2)	7.8	0.657	40	256	0.83	1.04	2.12	3.69	17.10	8.36
Lubrzanka R.^a^ (no. 8) (March 14, 2016)	7.9	0.604	48	165	<0.2	0.92	0.88	5.65	1.14	4.04
Lubrzanka R.^b^ (no. 9) (March 14, 2016)^d^	7.0	0.782	142	42	<0.2	0.59	14.1	2.02	7.71	32.8

For location of sampling sites see Fig. 1; concentrations of PO_4_^3–^ <0.1 mg/L; Alkal. – CaCO_3_ mg/L

*N* number of samples

^a^Upstream (control water samples) from the Wiśniówka mining area

^b^Downstream from the Wiśniówka mining area

^c^Discharge of AMD waters into the Silnica River

^d^Spills from tailings piles during heavy rains

**Fig. 3:**
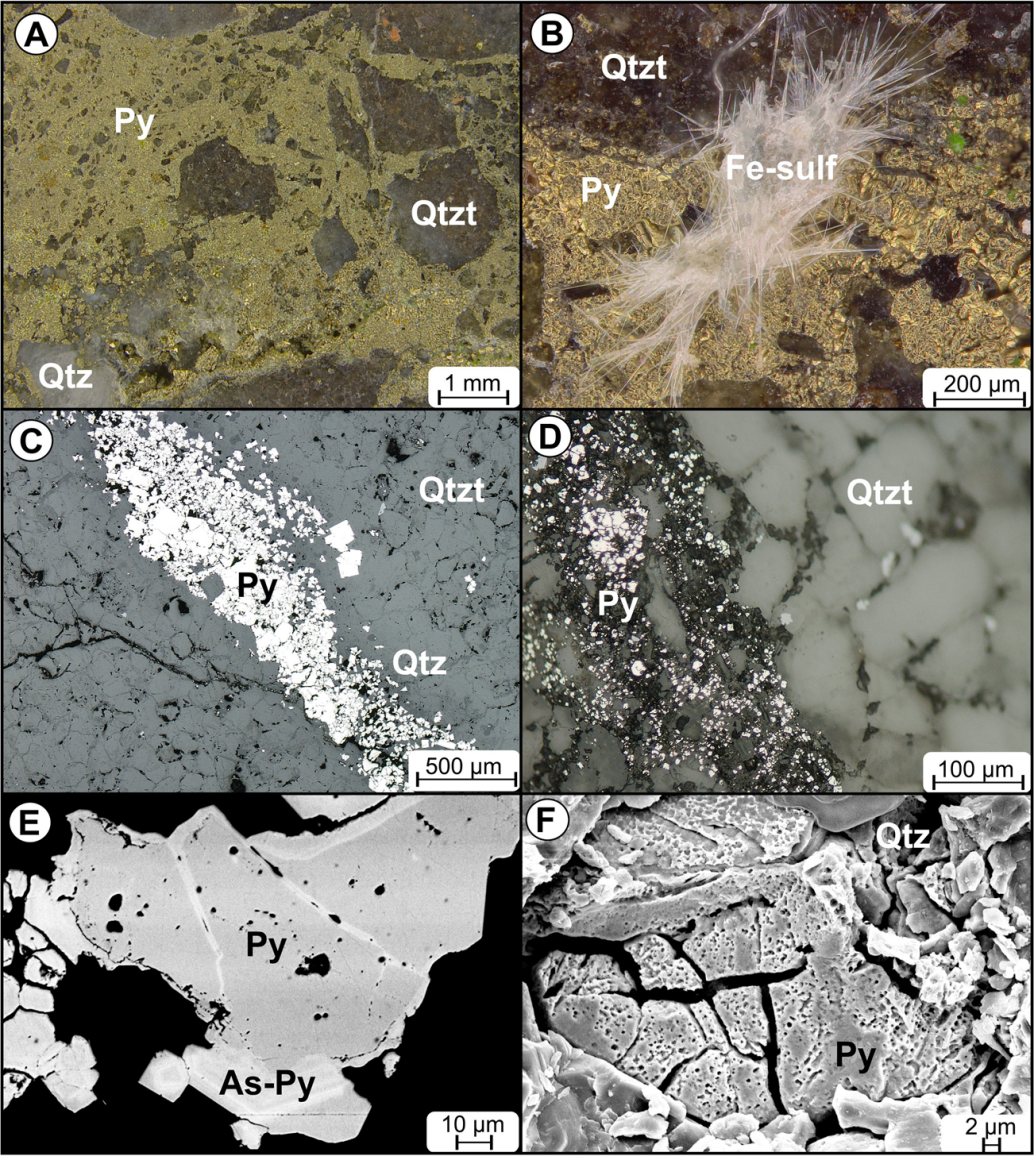
Stereoscopic micrographs of **a** tectonic quartzite-quartz tectonic breccia cemented with pyrite and **b** same tectonic breccia with needle-shaped secondary iron sulfates. Petrographic micrographs of **c** pyrite-quartz veinlet cutting quartzite, reflected light, 1 nicol, and **d** tectonic quartzite-quartz breccia cemented with crypto- and micro-grained pyrite, reflected light, 1 nicol. SEM images of **e** light gray As-rich pyrite forming veinlets and alternating bands with darker As-depleted pyrite (taken by L. Giro) and **f** weathered pyrite grain embedded in quartz veinlet (taken by L. Giro). *Py* pyrite, *As-Py* arsenic-rich pyrite, *Fe-sulf* iron sulfates, *Qtz* quartz, *Qtzt* quartzite

The petrographic study has shown that the principal source of AMD waters is oxidation of crypto- to fine-grained pyrite that occurs in the form of thin veins cutting fine-grained quartzites and quartzitic sandstones or scattered assemblages within most clayey shale interbeds, and additionally in the Podwiśniówka quarry as cements of tectonic quartz-quartzite breccias (Fig. 3a–d). The pyrite grains lack other sulfide mineral inclusions. However, the SEM-EDS and LA-ICP-MS study of Podwiśniówka mineralized rock samples revealed the presence of micro-veins and alternating bands of arsenic-rich pyrite (up to 8.2 % As) within an arsenic-depleted pyrite matrix (<1 % As) (Fig. 3e). The lack of As minerals and zonal distribution of arsenic may indicate that this element replaces both sulfur and iron in a pyrite crystal lattice, so not only sulfur as suggested by Kolker and Nordstrom (2001). Moreover, the pyrite comprises somewhat elevated concentrations of other elements (in parentheses maximum concentrations in mg/kg): Cu (1169), Ni (663), Zn (606), Co (121), Pb (107), and Cd (25) as indicated by the LA-ICP-MS study. The distinct determination coefficients (*R* ^2^) for two pairs Ni–As (0.95) and Co–As (0.68) may suggest that these two metals are linked to an As-rich pyrite variety as opposed to Cu which is uniformly distributed within the pyrite matrix. In contrast, Ni and Co substitute isomorphically for iron in a pyrite crystal lattice (Ribbe 1982). There is another significant constraint on the geochemistry of the acid waters examined. This is the lack of acid-consuming gangue and rock-forming minerals (except for subordinate muscovite displaying illitization) that have no attenuating effect on pyrite oxidation.

The oxidation of pyrite (Fig. 3b, f) has resulted in substantial lowering of pH, release of minor and trace elements from its crystal lattice, and remobilization of metal(loid)s, especially REE, from associated clayey shale interbeds. These combined processes have the potential to form acid waters that contain high concentrations of Al, As, Co, Cr, Cu, Fe, and Ni. The most interesting is the Podwiśniówka acid pit lake that shows a very low pH (2.2–2.5) and very high concentrations of SO_4_^2−^ (2720–5460 mg/L), Fe (545–1140 mg/L), Al (86.2 mg/L), As (9603–24,883 μg/L), Co (1317–3458 μg/L), Cr (753–2047 μg/L), Cu (6307–18,879 μg/L), Ni (1168–3127 μg/L), and REE (589–1341 μg/L). These contents exceed the permissible levels for industrial wastewater standards set by the Regulation of the Minister of Environmental Protection (2014) (see Table 1). Of the elements mentioned above, As, Fe_total_, Cu, Al, Ni, Cr, and Co concentrations exceed these limits by a factor of 96 to 249 (As), 54 to 114 (Fe), 13 to 38 (Cu), 29 (Al), 2 to 6 (Ni), 1.5 to 4 (Cr), and 1.3 to 3.5 (Co). By comparison, SO_4_^2−^ contents were 5 to 11 times higher than the set level of 500 mg/L. However, the high concentrations of As in the Podwiśniówka acid pit lake varying from 9603 to 24,883 μg/L, with a mean of 15,416 ± 5197 μg/L are of greater environmental concern (Tables 1 and 2). Along with strong acid pools, in which As contents reach 369,726 μg/L, these waters are among the most distinctive As-rich AMD surface waters throughout the world (Table 2).

In the most representative Podwiśniówka acid pit lake, which is presently not influenced by mining and processing operations, the lowest concentrations of SO_4_^2−^, Co, Cr, Cu, Ni, and the lowest values of TDS, salinity, and EC were noted in the winter of 2014 whereas the highest concentrations of Fe and Mn were observed in the summer of 2014. The highest levels of As and Ni were in turn found in the fall of 2014 and 2015. However, more data are needed for further discussion.

The three biggest acid water bodies (Table 1) showed diverse chemistry which is closely linked to mineralogy and petrology of exposed rock formations and different pyrite generations (study in progress). The lowest pH and the high metal(loid) concentrations in the Podwiśniówka acid pit lake water are due primarily to a greater amount of pyrite and presumably hematite and goethite. The REE-bearing minerals (gorceixite, florencite, xenotime, zircon) occur in clayey shale and tuffite interbeds of the Podwiśniówka quarry as shown by the previous studies (Migaszewski et al. 2007b; Migaszewski and Gałuszka 2010). For instance, some black clayey shale interbeds contain up to 454,66 mg/kg REE as opposed to pyrite which is substantially depleted in REE (22.02 to 90,65 mg/kg) (archival data). This is also reflected by high concentrations of REE (mean of 993 ± 282 μg/L) in the Podwiśniówka acid pit lake (Table 1). It is noteworthy that these concentrations are among the highest found in AMD streams, lakes, and ponds across the world (Table 2). These include acid mine effluents of the abandoned Zn-Pb mine of Santa Lucia in western Cuba (370–860 μg/L) (Romero et al. 2010) or stream waters of a Cu–Pb–Zn mining area in Metalliferous Hills, Italy (929 μg/L) (Protano and Riccobono 2002). It is noteworthy that the highest concentration of REE (29 mg/L) at a pH of 3.6 was reported in groundwater from the Osamu Utsumi uranium mine, Brazil (Miekeley et al. 1992).

The distinctly lower contents of As and trace metals were recorded in the Wiśniowka Duża acid pit sump (Table 1). The only exception was copper whose concentrations (878–2508 mg/L) exceeded the permissible limit by a factor of 2–5. In contrast, the SO_4_^2−^ concentrations (423 to 882 mg/L, mean of 575 ± 184 mg/L) varied around the permissible value of 500 mg/L (Regulation of the Minister of Environmental Protection 2014). Except for sulfate (494 to 648 mg/L, mean of 534 ± 64 mg/L), the Wiśniówka Mała acid pit lake water showed in turn the lowest concentrations of trace metal(loid)s (Table 1). Nonetheless, the levels of SO_4_^2−^ were higher than those recorded during 2005–2006 (108–192 mg/L) prior to washing of pyrite-rich quartzite aggregates from the Podwiśniówka quarry (Migaszewski et al. 2009).

It is interesting to note that the similar trace element concentrations to Wiśniówka Duża and Wiśniówka Mała acid pit waters were exhibited by historic tailings acid ponds (Table 1). Also, in this case, the SO_4_^2−^ concentrations were in the range of 256 to 976 mg/L due to heterogeneity of stockpiled mine waste material. The easternmost acid pond showed the lowest pH and the highest concentrations of sulfates and metal(loid)s, particularly Cu reaching 839 μg/L. This suggests that this portion of the tailings pile consists of Wiśniówka Duża waste material.

These three major acid water bodies of Podwiśniówka, Wiśniówka Duża, and Wiśniówka Mała have revealed some variations in sulfate and metal(loid) concentrations. The only exception was the pH that showed relatively stable values. Aside from Pb, Mn, As, and Zn in the Podwiśniówka acid pit lake and Wiśniówka Duża acid pit sump, the concentrations of remaining elements and SO_4_^2−^ did not show extreme variations (below a factor of 3). These may be induced by different influxes of pyrite oxidation products, changes in temperature, insolation, and precipitation patterns (rainfalls, droughts, ice cover), bacterial activity, secondary mineral transformations, sorption/desorption of trace elements, etc. (Acero et al. 2006; Leybourne and Johannesson 2008).

The extremely metal(loid)-rich acid pools (sampling point nos. 5–7 in Fig. 1) displayed the pH in the range of 1.7 to 2.8 and very high concentrations of SO_4_^2−^, Al, As, Co, Cr, Cu, Fe, Mn, Ni, and Zn (Table 2). Most of these parameters exceeded the set limits by a factor of several tens to hundreds, for instance concentrations of arsenic varied from 16,721 to 369,726 μg/L. It is interesting to note that REE concentrations were also very high reaching 6288 μg/L. However, these extreme values were lower than those (up to 8.15 mg/L REE) found in underground waters impacted by AMD in an abandoned uranium mining area of Eastern Thuringia, Germany (Merten et al. 2007).

There are no statistically significant correlations between the pH and element concentrations in the Wiśniówka AMD lakes, ponds, and pit sump. The best example is arsenic that does not exhibit any correlation with pH or SO_4_^2−^ concentrations. The other studies have also indicated relative mobility of this metalloid over a wide range of pH and redox conditions (e.g., Smedley and Kinniburgh 2002; Courtin-Nomade et al. 2005). However, the pH shows correlation with EC (*R* ^2^ = −0.86) and in turn EC with As (0.97) and Ni (0.88) whereas As with Ni (0.86). The highest determination coefficients (*R* ^2^ > 0.90) are noted for the following pairs: SO_4_^2−^ – Fe_total_ (0.97), Mn–Pb (0.97), Co–Sc (0.96), Al–Cd (0.95), Co–Ni (0.94), Co–Cu (0.93), and Ni–Sc (0.93). The strong correlations between As or Ni and EC may also point to a relationship of these two elements with dissolved and some colloid fractions (<0.45 μm). In contrast to As, Ni should not have a high affinity to amorphous hydrous ferric oxides under acidic conditions. This metal may show affinity with some clay minerals (illite, kaolinite, and mixed-layered illite-smectite) that were found in the sediment of the former Podwiśniówka acid pit pond (Migaszewski et al. 2008), but this issue needs further studying.

In contrast to the acid water bodies described above, the Lubrzanka and Silnica rivers upstream and downstream from the Wiśniówka mining area revealed the pH values in the range of 6.9 to 7.9 and simultaneously low concentrations of SO_4_^2−^ and trace amounts of metal(loid)s, including As (0.59–4.86 μg/L) and REE (0.604–5.625 μg/L). An insignificant drop in the pH and alkalinity, and a raise in concentrations of some metal(loid)s were noted during rainy season in March of 2016. This was caused by a small inflow of AMD waters as a result of long-lasting rainfall.

**Fig. 4:**
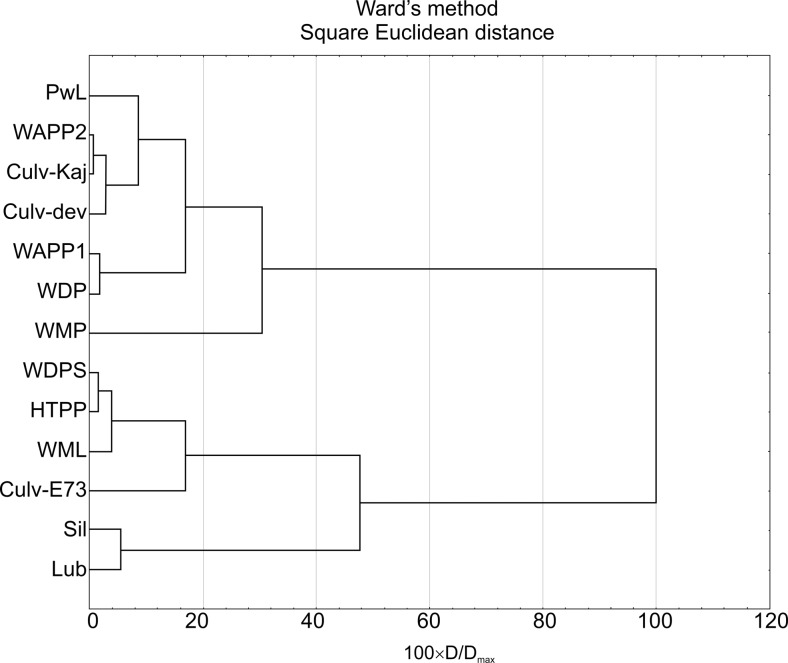
Spatial (site) dendrograms of AMD waters of the Wiśniówka mining area vs. Silnica and Lubrzanka rivers. *PwL* Podwiśniówka pit lake (no. 1 in Fig. 1), *WAPP2* Wiśniówka aggregate processing plant pool 2 (no. 7), *Culv-Kaj* culvert under railroad track in Kajetanów (no. 14), *Culv-dev* culvert under housing development (no. 13), *WAPP1* Wiśniówka aggregate processing plant pool 1 (no. 6), *WDP* Wiśniówka Duża pool (no. 5), *WMP* Wiśniówka Mała pond (not shown in Fig. 1, between nos. 3 and 13), *WDPS* Wiśniówka Duża pit sump pond (no. 2), *HTPP* historic tailings pile ponds (no. 4), *WML* Wiśniówka Mała pit lake (no. 3), *Culv-E73* culvert under highway E-73 (no. 12), *Sil* Silnica River downstream (no. 11), *Lub* Lubrzanka River downstream (no. 9). For location of sampling points, see Fig. 1

The element relationship between different acid pit water bodies and the Silnica or Lubrzanka rivers may also be evidenced by a spatial (site) variable dendrogram that groups these objects into two clusters at the linkage distance (Dlink/Dmax × 100) < 51 (Fig. 4): (i) Podwiśniówka lake (PwL), metal(loid)-rich acid pools (WAPP1, WAPP2, WDP), culverts (Culv-Kaj, Culv dev) (<15) and metal(loid)-rich acid pool (WMP) (<30), and (ii) Wiśniówka Duża pit sump (WDPS), Wiśniówka Mała lake (WML), and historic tailings pile ponds (HTPP) (<5) and culvert (<15). The acid pools of the first cluster form seeps at the foot of Podwiśniówka aggregate and waste material piles, hence their close relationship with geochemistry of the Podwiśniówka acid pit lake water. These seeps also flow down through two culverts (Culv-Kaj, Culv dev). The remaining AMD water bodies are in turn grouped together in the second cluster giving evidence for interrelated Wiśniówka Duża and Wiśniówka Mała mining and processing operations. Nonetheless, none of these two groups do exhibit any linkage to Silnica (Sil) and Lubrzanka (Lub).

### NASC-normalized REE patterns

The average measured LREE/HREE and NASC-normalized REE concentration ratios (HREE_NASC/_LREE_NASC_, La_NASC_/Yb_NASC_, Sm_NASC_/Yb_NASC_) are summarized in Table 4. In all these water samples, the measured LREE prevail over HREE with ratios in the range of 1.49 (Wiśniówka Duża pit sump) to 2.58 (Podwiśniówka acid pit lake). Nonetheless, the reverse relationship is observed when comparing their shale-normalized equivalents: the HREE_NASC_/LREE_NASC_ ratio varies from 1.76 (Podwiśniówka pit lake) to 3.52 (Wiśniówka Duża pit sump). The same relationship is observed between shale-normalized La/Yb and Sm/Yb ratios, for instance the La_NASC_/Yb_NASC_ ratio is in the range of 0.12 (Wiśniówka Duża) to 0.34 (Podwiśniówka) (Table 4). The lowest Sm_NASC_/Yb_NASC_ ratio (0.71) in the Wiśniówka Duża pit sump suggests that LREE are scavenged mostly by Fe- and Mn-oxide/oxyhydroxide and clay colloids (Sholkovitz 1995; Johannesson and Zhou 1999). However, experiments done on kaolinite and Na-montmorillinite have shown that the REE sorption is more complex and is controlled by the cation exchange capacity of clay minerals, pH, and ionic strength, for instance at higher ionic strength (0.5 vs. 0.025 M), the HREE were more sorbed than the LREE (Coppin et al. 2002). This means that the fractionation of REE between solid and solution phases still remains an unresolved issue.

**Table 4:** Average measured and NASC-normalized REE concentration ratios in AMD water bodies of the Wiśniówka mining area and adjacent rivers

Location	Pw pit lake (*N* = 6)	WD pit sump (*N* = 5)	WM pit lake (*N* = 5)	Historic tailings pit ponds (*N* = 6)	Silnica R. downstream (*N* = 1)	Lubrzanka R. downstream (*N* = 1)	Clayey shales Pw quarry (*N* = 9)
REE_(La–Lu)_ (μg/L)	829	174	95	214	5.62	0.80	348.54^a^
LREE_measured_/HREE_measured_	2.58	1.49	2.21	1.91	2.12	10.85	15.26^a^
HREE_NASC_/LREE_NASC_	1.76	3.52	2.34	2.74	3.57	0.86	0.50
La_NASC_/Yb_NASC_	0.34	0.12	0.32	0.17	0.52	0.79	2.76
Sm_NASC_/Yb_NASC_	1.97	0.71	1.25	1.00	0.41	0.47	2.56
Ce/Ce_NASC_^a^	0.99	1.19	0.96	0.96	0.42	0.96	1.15
Eu/Eu_NASC_^a^	1.38	0.92	1.17	1.07	0.65	1.27	1.74
Gd/Gd_NASC_^a^	1.46	1.34	1.45	1.37	1.53	0.92	1.15
Tb/Tb_NASC_^a^	1.30	1.40	1.41	1.42	1.39	1.12	0.82

^a^Excluding Lu concentration

**Fig. 5:**
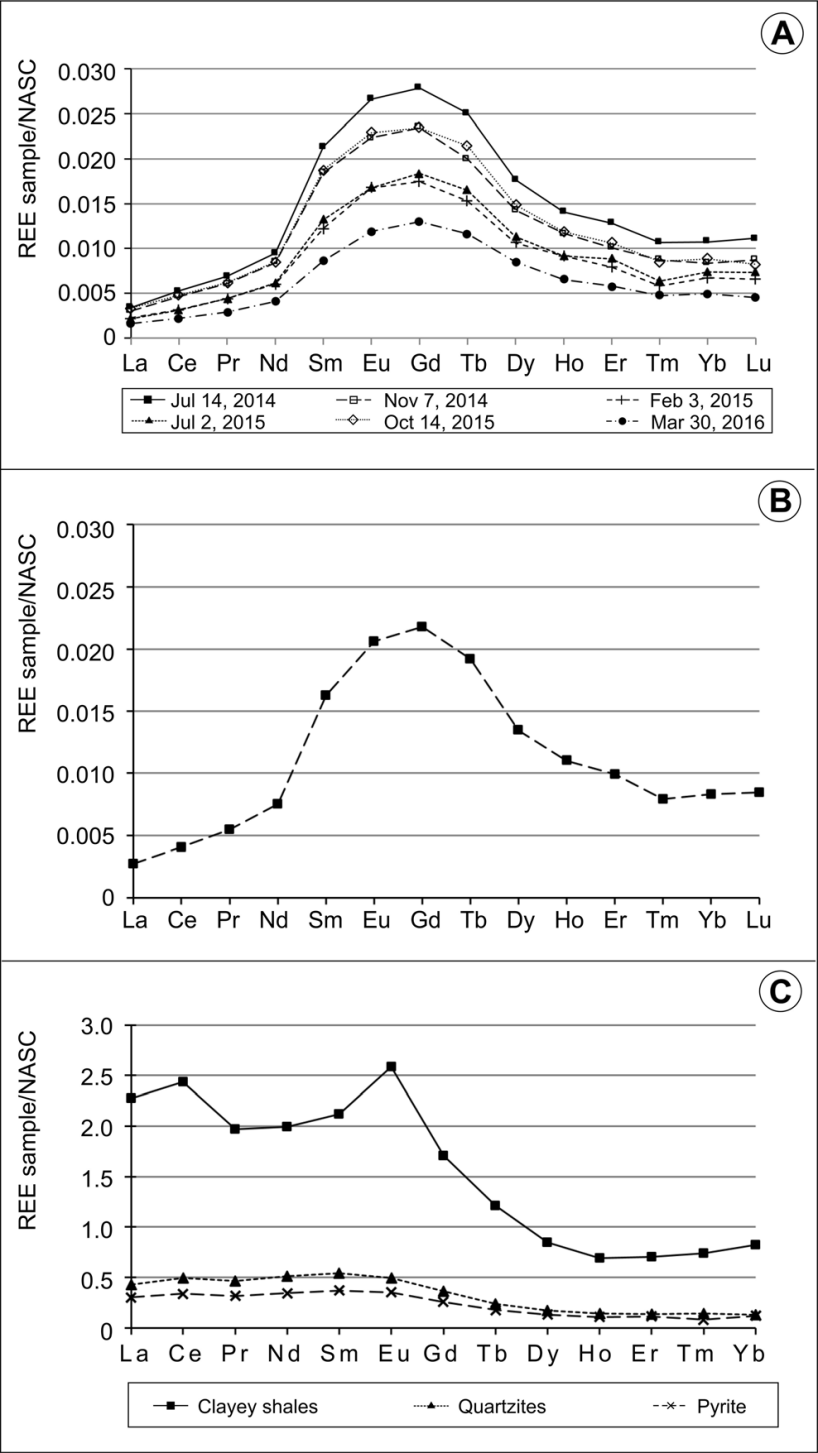
NASC-normalized REE concentration patterns of Podwiśniówka new acid pit lake water: **a** a series of six measurements and **b** mean value of six measurements, **c** patterns of rocks and pyrite (Migaszewski et al. 2014)

**Fig. 6:**
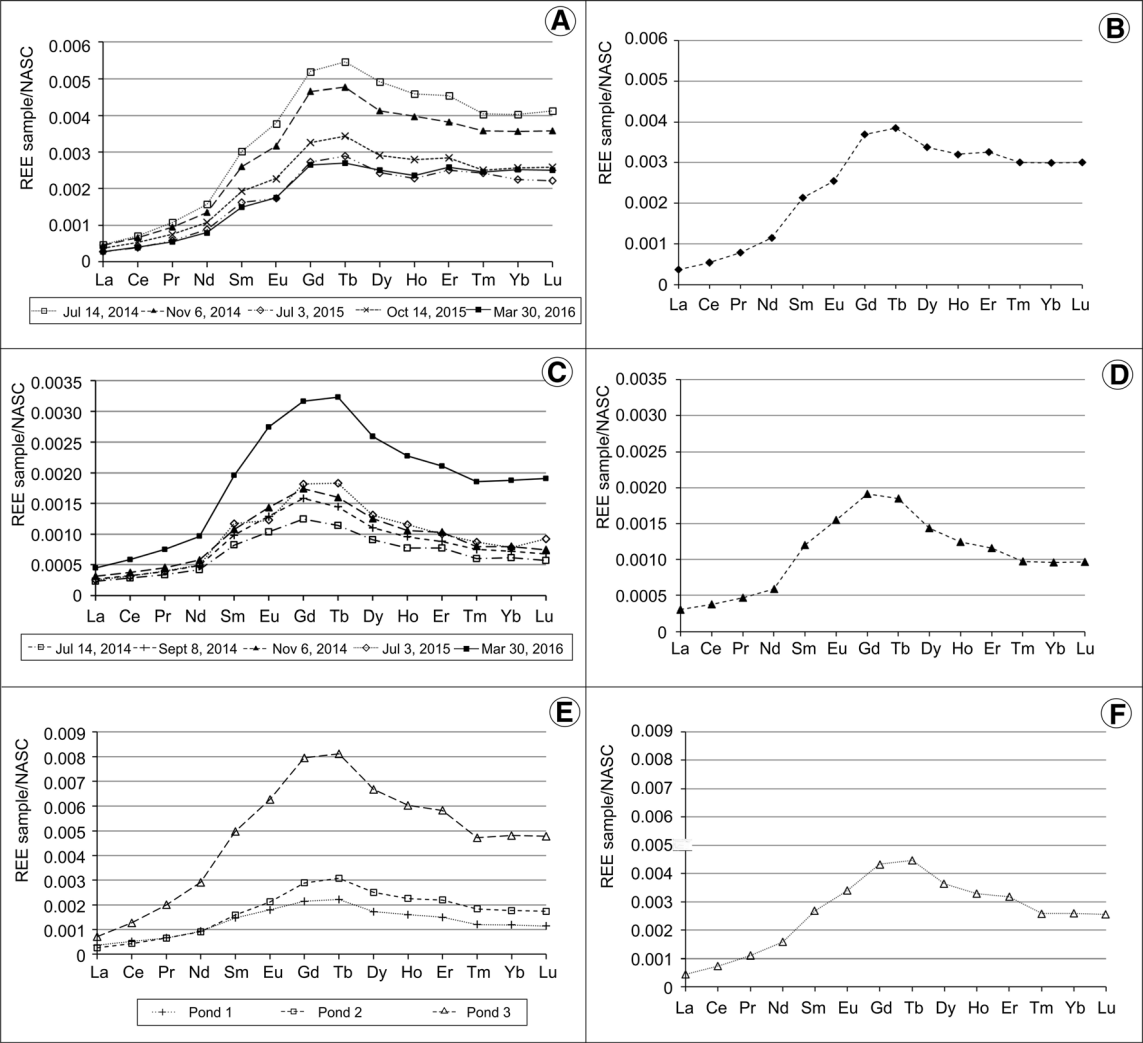
NASC-normalized REE concentration patterns of different acid water bodies: Wiśniówka Duża pit sump water: **a** a series of five measurements, **b** mean value of five measurements; Wiśniówka Mała acid pit lake (western part) water: **c** a series of five measurements, **d** mean value of five measurements; acid pond waters on historic tailings piles: **e** three selected ponds, **f** mean value of three measurements

The predominance of HREE over LREE is also evidenced by the normalized to NASC mean REE concentration patterns of Wiśniówka acid lakes and ponds (Figs. 5 and 6). This may be explained by preferential withdrawing of LREE from solution as a result of scavenging/adsorption by Fe- and Mn-oxide/oxyhydroxide colloids, poorly soluble secondary minerals (including jarosite), or organic matter-coated suspended detrital mineral particles (e.g., Gammons et al. 2003; Romero et al. 2010). Experiments document that despite considerable Fe(II) oxidation, REE remain in solution at a pH < 5.1, but in the range of 5.1–6.6, the HREE are removed from the water column compared to LREE and are partitioned into the ferric iron solid phases (Verplanck et al. 2004).

The Podwiśniówka acid pit lake water reveals a characteristic roof-shaped NASC-normalized REE concentration pattern with the prevailing MREE, especially Gd, Eu, and Tb (Fig. 5a, b). The predominance of these three elements is enhanced by the Gd/Gd_NASC_, Eu/Eu_NASC_, and Tb/Tb_NASC_ ratios: 1.46, 1.38, and 1.30, respectively (Table 4). Moreover, this convex-up REE profile suggests that MREE are either more easily leached from the host rocks or are more dissolved under the pH and redox conditions that prevail in AMD ponds and lakes (Leybourne et al. 2000). The MREE enrichment in AMD waters has been reported worldwide by many authors (e.g., Johannesson and Zhou 1999; Gimeno Serrano et al. 2000; Worrall and Pearson 2001; Zhao et al. 2007; Pérez-López et al. 2010; Grawunder et al. 2014). However, the results derived from these studies suggest that other explanations of this phenomenon must be also considered, for example dissolution of primary minerals—uraninite (McLennan 1994), apatite (Hannigan and Sholkovitz 2001), and pyrite (e.g., Grawunder et al. 2014)—and/or secondary minerals—Fe-oxyhydroxysulfates (Pérez-López et al. 2010) or Fe-Mn-oxides and oxyhydroxides (Johannesson and Zhou 1999).

The clayey shales show a distinct predominance of NASC-normalized LREE over HREE mean concentrations (*N* = 9) with a distinct positive Eu anomaly (Fig. 5c). The Eu/Eu^*^_NASC_ ratio amounts to 1.74. This is also enhanced by the HREE_NASC_/LREE_NASC_ ratio averaging 0.50. It is interesting to note that Podwiśniówka clayey shales and acid pit lake water do not reveal any Ce anomaly which is also evidenced by Ce/Ce^*^_NASC_ ratios averaging 1.15 and 0.99, respectively. This may suggest an invariable transfer of the REE patterns from these rocks to the pit lake water with insignificant modification by diverse solubility of REE-bearing minerals and different geochemical processes (sorption/desorption, dissolution).

The determination coefficients (*R* ^2^) between concentrations of individual REE and Mn, Fe, Al, and SO_4_^2−^ in the Wiśniówka AMD waters are presented in Table 5. It is interesting to note that Pr through Dy show very strong correlations (≥0.85) with Mn. Of the LREE group, Ce and La reveal distinctly stronger correlation with Al (*R* ^2^ = 0.85 and 0.86) than with Fe (*R* ^2^ = 0.38 and 0.35). The factor analysis for all the measured parameters does not exhibit isolated structures within the dataset. Al, La, and Ce have been related to factor 2. These results contradict those obtained by Gammons et al. (2005) who reported selective partitioning of HREE to Al oxyhydroxides. In contrast, the HREE and Sm have far greater affinities to Fe (*R* ^2^ = ∼0.75) than to Al (*R* ^2^ = ∼0.48). On the other hand, different studies have also shown that the REE may occur in the water as prevailing free metal cations (Leybourne et al. 2000) and sulfate complexes LnSO_4_^+^ (Johannesson and Lyons 1995). The last option must be considered for the Wiśniówka AMD waters due to the strong correlation of HREE with SO_4_^2−^ ions; *R* ^2^ varies from 0.70 (Gd) to 0.87 (Lu) (Table 5). This REE fractionation may be coupled at least partly with multiple adsorption-desorption reactions and/or ion exchange between the water column and precipitates, colloids, or secondary minerals as reported by other authors (e.g., Johannesson and Lyons 1995; Gimeno Serrano et al. 2000; Romero et al. 2010).

**Table 5:** Determination coefficients (*R* ^2^) between concentrations of individual REE and Mn, Fe, Al, SO_4_^2−^in the Wiśniówka AMD water bodies

REE	Mn	Fe_total_	Al	SO_4_^2−^
La	0.76	0.35	*0.86*	0.39
Ce	0.77	0.36	*0.85*	0.42
Pr	*0.85*	0.52	0.73	0.53
Nd	*0.88*	0.62	0.62	0.61
Sm	*0.89*	0.74	0.36	0.70
Eu	*0.90*	0.72	0.37	0.67
Gd	*0.89*	0.73	0.41	0.70
Tb	*0.88*	0.74	0.47	0.73
Dy	*0.85*	0.73	0.50	0.75
Ho	0.82	0.74	0.52	0.77
Er	0.79	0.74	0.52	0.79
Tm	0.75	0.74	0.52	0.80
Yb	0.72	0.77	0.46	0.83
Lu	0.68	0.80	0.41	*0.87*

Values showing the strongest *R* ^2^ (≥0.85) are italicized

In contrast, the Wiśniówka Duża acid pit sump exhibits the totally different step-shaped NASC-normalized REE concentration pattern with the most distinct predominance of HREE and positive Tb and Gd positive anomalies (Fig. 6a, b). This is confirmed by the Tb/Tb^*^_NASC_ and Gd/Gd^*^_NASC_ ratios, which are 1.40 and 1.34, respectively (Table 4). This also suggests that the younger Upper Cambrian beds (compared to the Podwiśniówka and Wiśniówka Mała quarries) must reveal different mineralogy and geochemistry, which is also evidenced by the lack of excessive amounts of As, Co, Cr, Fe, and Ni (see the “Geochemistry of waters section).

Like the Podwiśniówka acid pit lake, the Wiśniówka Mała acid pit lake shows nearly the same shale-normalized mean REE concentration pattern with the predominance of Gd and Tb (Fig. 6c, d). Both elements exhibit distinct Gd/Gd^*^_NASC_ (1.45) and Tb/Tb^*^_NASC_ (1.41) ratios. This overlaps the NASC-normalized REE profile of the former Podwiśniówka acid pit pond that was located at a higher mining bench compared to the current Podwiśniówka acid pit lake (Migaszewski et al. 2014). It is noteworthy that this pattern has changed since July 3 of 2015 due to the use of Wiśniówka Mała pit lake water for washing of crushed aggregate and dumping waste material from the Wiśniówka Duża (Fig. 6c, d). It is interesting to note that this change has not been accompanied by a distinct shift in the Wiśniówka Mała water chemistry.

The Ce/Ce_NASC_^*^ ratios in the acid reservoir waters varied from 0.96 (Wiśniówka Mała) to 1.19 (Wiśniówka Duża). If there is any anomaly, then it is rather a small positive one with 1.19. Except for Podwiśniówka, the Eu/Eu^*^_NASC_ ratios varied from 0.92 (Wiśniówka Duża) to 1.17 (Wiśniówka Mała).

The historic tailings acid ponds and intermittent strong acid pools display the NASC-normalized REE patterns bodies (Fig. 6e, f) similar to those of Podwiśniówka and/or Wiśniówka Duża water. Moreover, they generally exhibit intermediate NASC-normalized REE concentration ratios with the predominance of Tb/Tb^*^_NASC_ (1.42) and Gd/Gd^*^_NASC_ (1.37) (Table 4). Some variations in the NASC-normalized REE patterns and ratios depend on the localization of ponds on historic tailings which are built of mine and processing wastes derived from different sources. This issue is discussed in more detail in the “Element fingerprints of mining operations” section.

### Element fingerprints of mining operations

The study area is a unique AMD site where only rocks (quartzites) are quarried whereas pyrite constitutes only subordinate mineralization of no economic value. This is in contrast to most AMD sites throughout the world which are closely linked to metal ore or coal deposits (e.g., Monterroso et al. 1998; Plumlee et al. 1999; Hudson-Edwards et al. 1999; Nordstrom and Alpers 1999a, b; Protano and Riccobono 2002; Gammons et al. 2003; Zhao et al. 2007; Romero et al. 2010; Migaszewski et al. 2015). Another distinguished feature of the Wiśniówka mining area is a lack of other sulfide minerals and effectively buffering gangue minerals and rocks. As discussed in the previous two sections, the three acid pit water bodies exhibit different chemistry that generally correlates with the geologic makeup of the quarries. This also finds its reflection in the specific roof- and step-shaped (Podwiśniówka vs. Wiśniówka Duża) NASC-normalized REE concentration patterns and characteristic enrichment of Podwiśniówka rock series (and waters) in As, Co, Cr, Cu, and Ni that enable identification of potentially hazardous places.

The Podwiśniówka tailings and crushed aggregate stockpiled at a depository site near the Wiśniówka Mała quarry and near the Wiśniówka aggregate processing plant give rise for concern. These piles are a source of seeps forming strong acid and As- and metal-rich pools. These in turn are washed away by rainfalls outside of the mining area occasionally jeopardizing the neighboring river systems. Similar potential threats posed by seeps to the environment have been studied in other AMD sites using mining-related metal(loid)s (e.g., Mighanetara et al. 2009). The REE patterns were also employed to identify seepage water from historic tailings piles as a source of contamination of valley sediments at Ronneburg mining area (e.g., Merten et al. 2005).

The comparison of geochemical signatures of historic tailings ponds with those of the three acid pit water bodies has indicated that these tailings piles are built of mine wastes derived primarily from extraction of Wiśniówka Mała and Wiśniówka Duża quartzites. In addition, the chemistry of some pools that form in the Podwiśniówka and Wiśniówka Duża quarries provides information on unexposed pyrite occurrences that may locally occur within the intensively folded and faulted Wiśniówka massif. This may give a clue to the future mining operations in a tectonically affected area.

### Interactions of mine drainage with the environment

The results of this study show an insignificant impact of untreated AMD waters from the Wiśniówka Duża pit sump and Wiśniówka Mała lake on the neighboring river systems due to the presence of carbonate rocks (limestones, marly shales, limestone conglomerates) in the Silnica River bed and partly in an upper course of the Lubrzanka River. This is the reason why the waters of these streams have an effective buffering capacity (Table 3). For example, close to the confluence of a ditch (draining the Wiśniówka Duża pit sump; sampling point nos. 5 and 12) and the Silnica River downstream (sampling point no. 11), the pH increased from 3.3 to 6.9 and the concentrations of SO_4_^2−^ and Cu dropped from 423 to 134 μg/L and from 878 to 86 μg/L, respectively. The same downward trend was also noted for the REE (from 127.5 to 5.6 μg/L), Al (from 18.9 to 0.44 mg/L), Fe (from 10.6 to 0.4 mg/L), and As (from 25.3 to 2.2 μg/L). Another significant issue is the presence of exposed and unexposed piles derived from former mining operations, especially from the Podwiśniówka quarry, which still contribute to elevated metal(loid) concentrations (of which arsenic is of greatest concern) in intermittent strongly acidic pools.

Unfavorable localization of the Wiśniówka mining area, i.e., a dismembered mountain range reaching 100 m in relative elevation and the proximity of villages, makes it difficult to utilize abiotic or biotic remediation strategies (e.g., Banks et al. 1997; Plumlee and Logsdon 1999; Coulton et al. 2003; Johnson and Hallberg 2005; Runkel et al. 2009). In addition, the lack of flat areas for construction of large biotic aerobic ponds or wetlands and (an) aerobic plots has forced the mining company to use other options of AMD water remediation, including anoxic limestone drains, impermeable barriers to potential acid seeps, lime addition, and revegetation of some tailings piles.

No action has in turn been taken against the Podwiśniówka acid pit lake. This part of the quarry is naturally watertight and to date has not posed a threat to the adjacent river systems or suspended local aquifers as indicated by the previous studies of farmer’s well waters (Migaszewski et al. 2014) and current monitoring. The only solution is to isolate the major part of the reactive pyrite mineralization zone by covering it with clays and soil, grassing over and planting with trees. Although currently the Podwiśniówka acid pit lake does not jeopardize the local underground water system, this site needs constant monitoring to evaluate its potential impact on the neighboring environment especially during long periods of rainfalls.

## Conclusions

The following conclusions can be drawn from the hydrogeochemical data derived from this study:

1. Different shale-normalized REE concentration patterns from the Podwiśniówka acid pit lake (roof-shaped with Gd, Eu and Tb positive anomalies) and the Wiśniówka acid pit sump (step-shaped with a strong predominance of HREE_NASC_ over LREE_NASC_) can be used along with some metal(loid)s as geochemical signatures of mining operations and their potential influence on the environment.
2. Due to fractionation of the REE during weathering, leaching of dissolved and suspended fractions, and geochemical interactions in the acid water bodies, the individual REE show greater affinities to Mn, HREE to Fe and SO_4_^2−^, and La and Ce to Al.
3. The chemistry of some acid pools provides information on unexposed pyrite occurrences within the intensively folded and faulted Wiśniówka massif. This may give a clue to the future mining operations in a tectonically affected area.
4. The acid water bodies of the Wiśniówka mining area currently show a minor impact on the surface and underground water systems. However, the results derived from this study also indicate that the Podwiśniówka acid lake and acid pools should be protected against accidental surficial runoff or leachate by constructing earth barriers or using naturally isolated depressions. Moreover, these “hot spots” needs constant monitoring to evaluate their impact on the neighboring environment.

The obtained data enable mine planners to better understand, anticipate, and mitigate potential environmental hazards. These have also been useful for working out realistic estimates and plans for quartzite extraction and processing, as well as for developing remediation and monitoring programs.
